# Digital artifacts reveal development and diffusion of climate research

**DOI:** 10.1038/s41598-022-17717-8

**Published:** 2022-08-19

**Authors:** Bia Carneiro, Giuliano Resce, Tek B Sapkota

**Affiliations:** 1 Centre for Social Studies, University of Coimbra, Colégio de S. Jerónimo Apartado 3087, 3000-995 Coimbra, Portugal; 2 Department of Economics, University of Molise, Via Francesco De Sanctis, 1, 86100 Campobasso, CB Italy; 3 International Maize and Wheat Improvement Center (CIMMYT), México-Veracruz, El Batán Km. 45, 56237 México, Mexico

**Keywords:** Climate-change adaptation, Climate-change mitigation, Climate-change policy

## Abstract

Research for development organizations generate tremendous amount of accessible knowledge, but given their scale, time and resource constraints, the impact of outputs is not systematically analyzed. This is because traditional bibliometric analyses present limitations to synthesize accumulated knowledge and retrofitting indicators to historical outputs. To address these shortcomings, this study proposes an integrated, web-based approach to systematically analyze the production and diffusion of knowledge from large-scale research programs, using climate research of the International Maize and Wheat Improvement Center (CIMMYT) as a case study. Our analytical framework employs text mining, social network analysis and hyperlink analysis to an unstructured mass of publicly available digital artifacts such as institutional repositories, citation databases, and social media to uncover narratives, dynamics, and relationships. Findings show CIMMYT’s climate research is strongly incorporated into a holistic systems approach and that the institution is actively engaged in knowledge exchanges with key actors from the scientific, development and public policy communities. The proposed analytical framework establishes an effective approach for research for development organizations to leverage existing online data sources to assess the extent of their knowledge production, dissemination, and reach.

## Introduction

Climate change directly affects food production and food systems, but the way we produce our food also has direct consequences to climate change^1^. Scientific research and innovation have progressively explored the complex interactions between food systems and climate systems to understand how climate change affects food production, and how to manage the impacts of food systems on climate change^2^. Scientific research institutions play a key role in knowledge generation, with non-profit research-for-development organizations among the key actors involved in the interface between knowledge and action^3^ and contributing at all stages of the research cycle: they have a central role in resource mobilization and in the generation, utilization, and management of knowledge^4^.

As the availability and accessibility of information in contemporary societies becomes a main driver of social transformation, in addition to being discovered, knowledge needs to be diffused^5^. Funders increasingly require evidence of the value of their research investments to society, and policymakers increasingly harness scientific evidence to support sustainable development policies^3^, engendering widespread interest in assessing the dissemination of knowledge arising from research-for-development programs^6^.

Research-for-development organizations are often unable to systematically assess the significance and impact of their outputs due to extensive research portfolios, time and resource constrains. Therefore, the dynamics of knowledge dissemination about strategic themes are not fully understood. A clear framework that monitors progress while accounting for the complexities of research-for-development remains a major challenge, as metrics traditionally employed to evaluate scientific production present limitations in assessing reach beyond the research community^7^. Additional obstacles include synthesizing the accumulated information spread not only across thousands of internal and external documents^8^, but also across various communications channels and platforms, and retrofitting indicators to historical research, as concepts and terminologies tend to evolve over time^9^.

The integration of digital research methodologies^10^, with big data analytics and machine learning techniques offers an innovative and comprehensive approach to deal with such challenges. Studies demonstrate the potential for leveraging on underexplored sources of information produced within organizations, such as project documentation, institutional communications or research outputs, as well as on digital artifacts^11^ available across the web, to generate knowledge about strategic issues that may be difficult to observe using traditional surveys or manual stocktaking. Examples include big data architecture frameworks for organizational data management^12^; the impact of real time data capturing and processing on government operations and policy making^13^; machine learning models to accelerate evidence generation that supports achieving the Sustainable Development Goals^14^; the development of platforms that collect real time data to monitor human societal-scale behavior and beliefs at a global scale^15^; and the application of digital methods to assess program influence and reach^7^, among others.

Focusing specifically on diffusion of research outputs, it has been shown that web tools like social media, blogs, or social bookmarking platforms, provide the possibility to construct innovative metrics to gauge scientific impact and influence^16^. The standard quantitative approach for measuring the diffusion of research outputs is based on bibliometric measures (such as Impact Factor or H-Index) that have been increasingly successful and are certainly valuable since they reveal not only the number of outputs produced by researchers but also the number of times these outputs have been cited, contributing to knowledge generation. However, bibliometric assessment of outputs such as scientific publications, reports, and other knowledge products do not capture the less explicit forms in which science influences decision making^5^.

This paper contributes to the efforts to broaden the evaluation of scientific contributions by proposing a multimodal analysis that leverages digital artifacts such as digitized research outputs, the web, and social media to map the creation, dissemination and reach of research through big data analytics and machine learning. As limited studies have focused on food systems and climate change research impact evaluations, we apply our framework to assess the climate science knowledge generated by the International Maize and Wheat Improvement Center (CIMMYT).

CIMMYT is a non-profit, international agricultural research for development organization that has been involved in extensive research on climate change adaptation and mitigation in agriculture, particularly in maize and wheat-based production systems across different regions. Funded by bilateral donors and by research programs of the Consultative Group on International Agricultural Research (CGIAR), the institution’s climate focused research aims to help farmers adapt to shocks while producing more food and reducing emissions, where possible.

The strong climate component of CIMMYT’s research, particularly in the last two decades, has generated several research outputs relevant to adaptation and mitigation actions in agriculture. Due to the limitations discussed, the Center lacked a consistent framework from which to monitor progress against its strategic climate agenda. This research aims to answer the following questions: what has been CIMMYT’s research for development focus within the food production-climate change nexus? How have CIMMYT’s climate-related knowledge products been disseminated across different communities (scientific, policy, practitioners, etc.) and geographies? How and with whom is CIMMYT engaging in the broader network of climate change research and action?

Building our analytical framework based on the Digital Methods perspective^10^ we systematically assessed CIMMYT’s climate research portfolio and assessed its engagement within and beyond the scientific community to uncover the institutional process of knowledge diffusion. The mixed methods approach employed text mining, hyperlink analysis and network analysis to identify and classify the research outputs generated by CIMMYT, map the distribution of knowledge products, and uncover networks of collaboration and dialogue among institutional actors.

The rest of the paper is organized as follows: section two presents our methodological approach and data collection processes, section three describes the results, section four discusses the implications for scientific evaluation, and section five concludes with some recommendations and forward-looking remarks.

## Data and methods

With over 60% of the world’s population connected to the internet^17^, its most prominent element, the World Wide Web, plays a crucial role within the technological infrastructure of society through the establishment of new social practices and new forms of knowledge exchange^18,19^. The continuous transformation of information technologies in the digital era have expanded the reach of communications tools to all aspects of social life through networks that are increasingly integrated into existing offline practices and social relationships^10,20^.The internet is effectively recognized as a flourishing space for research on social relations, spawning several approaches that advocate going beyond determining how much of society is online to researching cultural and social transformations via the internet^10,21^.

**Figure 1:**
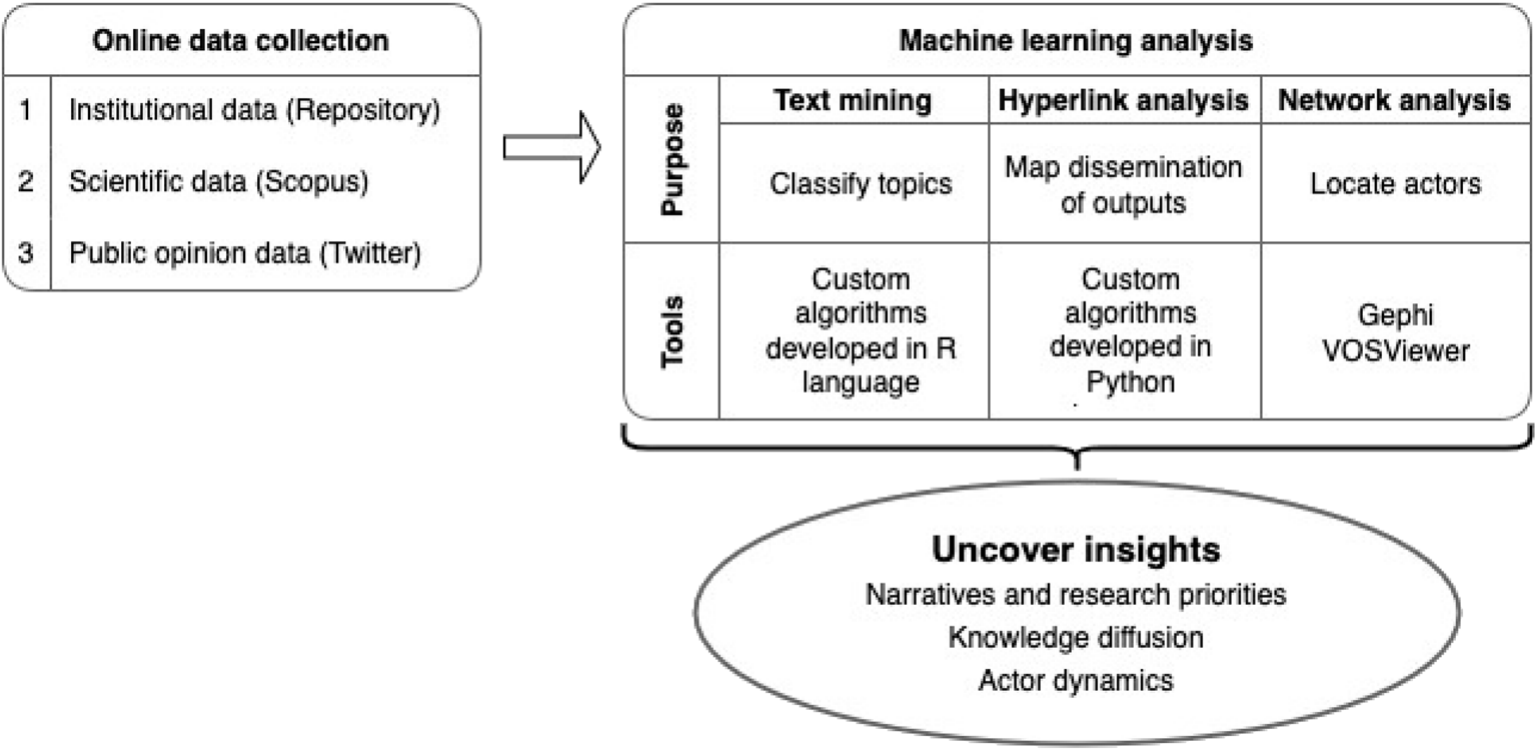
Analytical framework for digital research to assess knowledge flows of scientific research.

A growing body of literature has established the web and social media as dimensions that represent wider public debates, information exchanges, and engagement on various topics, such as climate^22,23^, politics^24^, social movements^25^, or health crises and misinformation^26,27^. Based on these notions, the analytical framework we propose aimed to assess the discourse and the knowledge flows of CIMMYT’s climate research by considering online representations and interactions as evidence of broader strategic engagement. We begin by identifying, collecting and processing the appropriate, publicly available digital artifacts^11^ (such as digitized research outputs, online content, social media). We then apply a data-driven, mixed methods approach that employs text mining, network analysis and hyperlink analysis to uncover dynamics, narratives, and relationships, as illustrated in Fig. 1.

This framework was used to assess CIMMYT’s work in the food production-climate change nexus, focusing on the following questions: What has been CIMMYT’s research for development focus within the food production-climate change nexus? How have CIMMYT’s climate-related knowledge products been disseminated across different communities (scientific, policy, practitioners, etc.) and geographies? How and with whom is CIMMYT engaging in public conversations about climate change research and action?

The next sections describe the specific datasets prepared and techniques employed in the analysis.

### Datasets

CIMMYT’s digital repository was selected as the central source for its research outputs and institutional publications. Upon testing a few combinations, the repository was queried for the keywords “climate” and “clima”, which resulted in 2,463 publications, including both scientific and communications products, disseminated between 1960 and 2021. These knowledge products comprise 46% of the entire database of documents, enabling broad coverage for text mining the corpus of abstracts for relevant topics. A custom algorithm was developed in Python language to scrape query results and extract available metadata. For each record, the information collected included: publication title, publication type, authors, abstract, year, publisher, and URL (i.e., the web address).

Scientific production was assessed through peer-reviewed publications indexed in the Scopus abstract and citation database^28^. A query was performed to identify indexed publications in which authors declared affiliation to CIMMYT. It resulted in 3,612 items published between 1974 and 2021. Full citation data and metadata were exported, including publication title; journal title, volume, and issue; authors; affiliations; keywords (author and indexed); funding details; abstract; year; DOI and URL; and references.

To uncover the broader public dialogues that CIMMYT has engaged in, the social media platform Twitter was selected. Twitter is widely recognized as an important forum for institutional communications, and in the literature that has assessed it in connection to online climate dialogues and action, it is considered an important “source for climate change information-exchanges”^22^. Its specific affordances as a real-time, topic-driven platform also make it suitable for detection of trends, to explore discourse dynamics^29^ and interactions with various stakeholders.

A custom algorithm was developed in Python language to search Twitter for publicly available tweets that either mentioned the institution’s profile (@cimmyt) or that contained the hashtag #CIMMYT between 2009 and 2021. We collected a total of 69,027 tweets in the period between 2009-02-06 and 2021-04-12. For every tweet, data extracted includes: time of publication, text of tweet, hashtags, mentions, number of favorites, number of replies, number of retweets, and URL of tweet.

For each dataset, text was extracted from its source and the corpus of analysis was prepared using functions from the R package “tm”^30,31^: punctuation, stop words (i.e., in English, words like “the”, “is”, “of”, etc.), and numbers were removed.

### Text mining

Text Mining is broadly defined as an approach that transforms large amounts of unstructured text from sources such as articles, reports, web pages, or social media posts and comments into structured and normalized data, which is then analyzed for implicit, previously unknown trends through machine learning, statistics and linguistics^32^.

Text mining was employed to derive insights on CIMMYT’s research outputs about climate change adaptation and mitigation, and to classify and quantify the relationship between these and other strategic topics in the data. A crucial step for the analysis was the development of a custom taxonomy that identifies key terminology against which we could map text from the data sources. Taking guidance from CIMMYT experts, we constructed a framework comprising two overarching themes: cross-cutting topics cover the strategic themes mainstreamed into CIMMYT’s research; climate-focused topics were identified to reflect specific techniques and technologies researched by the institution (the full list of 45 topics can be found in Supplementary Table A1 online).

A central element of textual analysis is semantic association, as terms specified in the taxonomy may be represented differently in the various sources. Expanding taxonomy terms to identify similar words improves the ability to uncover patters in the corpora. Given CIMMYT’s agriculture focus, a custom term expansion solution was developed using AGROVOC, the Food and Agriculture Organization’s (FAO) comprehensive, open-source, multilingual vocabulary^33^. Its structured collection consists of more than 37,000 terms covering FAO’s areas of interest, such as food, nutrition, agriculture, fisheries, forestry, environment etc. Vocabulary is hierarchically organized in 25 overarching concepts and is available in up to 37 languages, enabling multilingual analysis.

For each topic, the terminology used by CIMMYT was matched to AGROVOC (the full list of 45 matched terms can be found in Supplementary Table A1 online). The corresponding AGROVOC definition was extracted in a JSON file, and a custom algorithm was developed to detect and classify the related terminology within the text of the various data sources. For each document $\mathrm{j}$ (descriptions, abstracts, tweets), we quantified the presence of a term $\mathrm{i}$ defined in AGROVOC, as shown in Eq. (1). The list of country names from the *world.cities* database in the ‘maps’ R code^34^ was taken to develop an algorithm that identified countries mentioned in the text.

1 $$\frac{\frac{\sum \left({\mathrm{Words}}_{{\mathrm{Document}}_{\mathrm{j}}}\in {\mathrm{Words}}_{{\mathrm{AGROVOC}}_{\mathrm{i}}}\right)}{\sum {\mathrm{Words}}_{{\mathrm{Document}}_{\mathrm{j}}}}}{\underset{\mathrm{i}}{\mathrm{max}}\left[\frac{\sum \left({\mathrm{Words}}_{{\mathrm{Document}}_{\mathrm{j}}}\in {\mathrm{Words}}_{{\mathrm{AGROVOC}}_{\mathrm{i}}}\right)}{\sum {\mathrm{Words}}_{{\mathrm{Document}}_{\mathrm{j}}}}\right]}$$

### Hyperlink analysis

An approximation of how the organization’s knowledge products have been disseminated globally was explored through an adaptation of hyperlink analyses approaches^35^ that aim to identify actors and geographies of particular issues as represented by hyperlink connections. We adjusted the approach to explore knowledge diffusion and uncover information flows.

The list of publications extracted from CIMMYT’s repository served as the root source from which to uncover how they have spread across the web. For every unique publication, a search for all the web pages hyperlinking to it was carried out by means of web scraping algorithms. This led to the identification of the web pages hyperlinking to every item of the considered list. Three commercial search engines were queried: Google, Bing, and DuckDuckGo. Due to limitations to searching directly for hyperlinks in these engines, the algorithm developed searches for the presence of all words in the title of the research output and for a string of the disseminated URL.

The approach works well for publications with specific titles, but broad titles can lead to ambiguous results. The scraping algorithm included a mechanism to detect such ambiguous results. In the final output, every extracted record contains: title of original publication; URL of web page containing a reference to the publication; domain of the URL (e.g., repository.cimmyt.org from https://repository.cimmyt.org/handle/10883/20129 ); Top Level Domain (TLD) of the URL (e.g. .org or .com); and metadata such as publication type, year, authors, and publisher. This output was then analyzed for trends.

### Network analysis

Using the data collected from Twitter and its specific affordance of enabling direct conversations among users through the @mention, we performed a network analysis to place CIMMYT within a broader network of actors engaged in dialogue exchanges about the food production-climate nexus. A mentions network^36^ enables the visualization of relational data organized as matrices, where @mentions within the tweets are the nodes and their relations are the lines connecting pairs of nodes; accounts are connected if they mention one another. The weight of this connection is calculated from the number of mentions by the same account, capturing not only the presence of a connection, but also the strength of the connection as a measure of significance.

A subset of approximately 5 thousand tweets containing climate-related content was prepared, and a matrix containing the accounts scraped and the accounts mentioned by them in the tweets was constructed. The open-source software Gephi^37^ was used to import the matrix and plot the network graph. The force-directed algorithm Force Atlas 2 was applied to show the spatialization of nodes by mapping the proximity and the authority of categories in relation to each other^38^. A modularity algorithm^39^ was applied to identify “communities”, or clusters—as represented by nodes that are more densely connected than to the rest of the network, and which were colored accordingly.

The dynamics of scientific contributions were explored from the Scopus dataset. VOSviewer^40^, an open-source bibliometric analysis software, was used to import the Scopus data and create network visualizations based on the declared affiliations of publication authors and the countries of their respective institutions.

## Results

### Text mining: uncovering trends in CIMMYT’s climate research agenda

Leveraging on textual data from CIMMYT’s digital Repository, Scopus, and Twitter, we systematically reviewed trends in the center’s research priorities in relation to climate change and determined how the resulting knowledge products have been communicated to the public.

Text mining uncovered trends and insights from three perspectives: at the institutional level, at the scientific production level, and at the broader public outreach level. Descriptive analysis of the metadata from the Repository shows that most items available are scientific outputs, such as articles and books, but there are also hundreds of reports, presentations, handbooks, brochures, and other communications products. Besides their availability in the Repository, publishers of the research include scientific journals, funders and partners, as summarized in Supplementary Figs. B1–B3 online. The country detection algorithm indicates a focus on Mexico and India as the most frequently mentioned countries, followed by Bangladesh, Ethiopia, Kenya, Pakistan, and Nepal (full country distribution can be found in Supplementary Fig. C1 online).

Analysis from Scopus shows that while CIMMYT has published extensively in journals from the fields of agricultural and biological sciences, there have also been many research outputs in biochemistry, genetics, and molecular biology, as well as in environmental and social sciences. Mexico and India are once again the most frequently detected countries, followed by Zimbabwe and Ethiopia (full country distribution can be found in Supplementary Fig. C2 online).

Twitter data provides a broader perspective of public, non-scientific perceptions of CIMMYT’s work and how its expertise is recognized by the wider community within this subject area. The geographical coverage of conversations that CIMMYT has engaged with is widespread in this dataset, as almost all countries worldwide have been mentioned in one way or other in Twitter conversations, with Mexico and India still the most frequent, followed by Kenya, Ethiopia, and Pakistan (full country distribution can be found as Supplementary Fig. C3 online).

**Figure 2:**
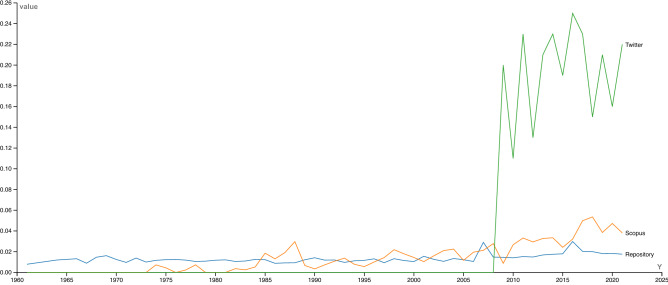
Time trends for the average prevalence of “climate change” detected in the datasets.

To understand the historical evolution of CIMMYT’s climate research agenda, Fig. 2 presents the time trends for the topic ‘Climate change’ detected across the three data sources. Beyond the presence or absence of a topic, the classification algorithm also quantified the prevalence normalized on a scale from 0–1, where 1 represents the maximum value detected over the period of analysis.

The Repository timeline shows the institution has historically addressed the effects of climate change in agricultural production, but scientific outputs indexed in Scopus reveal an increase since the turn of the millennium. The time span for the Twitter dataset is much shorter than for the other sources due to the platform’s founding in 2006 and CIMMYT’s profile creation in 2009, when the issue of climate change was already widely present in public debates. The Twitter timeline indicates that climate change has been priority issue in CIMMYT’s conversations, with significant coverage since the institution joined the platform. The time trends for individual topics can be found in Supplementary Figs. C1–C3 and D1–D3 online.

Considering only content from which the topic ‘Climate change’ was detected, Table 1 presents the frequency distribution of the other cross-cutting topics detected in corpora from the various sources. It shows that climate-related materials disseminated through the Repository focused primarily on ‘Farming systems’, ‘Food security’, ‘Technology transfer’, and ‘Innovation’. The Scopus corpus indicates two thirds of CIMMYT’s scientific production focused on the relationship between climate and food security, followed by ‘Farming systems’, ‘Stress’ (i.e., physical, or biological responses to a stressor) and ‘Yields’. Twitter’s topic distribution is more dispersed, but still aligned with CIMMYT’s emphasis on ‘Food security’ and ‘Farming systems’, as well as on ‘Innovation’ and ‘Technology transfer’, both topics prevalent in the Repository.

**Table 1:** Distribution of cross-cutting topics identified in the datasets.

Cross-cutting topic	Repository (%)	Scopus (%)	Twitter (%)	Overall (%)
Capacity development	7.7	19.6	80.7	14.8
Diversification	0.9	1.7	21.5	1.9
Enterprises	5.7	24.6	90.4	15.8
Farming systems	31.5	37.8	98.5	36.4
Food security	26.3	66.0	98.5	43.5
Gender equity	3.8	0.9	32.6	1.8
Health	6.5	16.7	84.4	13.2
Innovation	16.7	27.8	97.8	23.8
Livelihoods	1.6	2.4	52.6	3.9
Mixed cropping	2.3	4.9	45.9	5.0
Nematoda	0.4	0.5	4.4	0.6
Nitrification	1.0	7.5	48.1	5.2
Nutrition	2.8	4.3	72.6	6.1
Participation	2.8	4.1	68.1	5.8
Policies	0.9	4.2	55.6	4.2
Services	3.7	5.3	71.1	6.9
Socioeconomic development	4.1	7.8	65.9	7.8
Stress	13.2	33.5	85.9	23.4
Technology	9.3	17.9	90.4	15.6
Technology transfer	16.9	23.1	97.0	22.3
Yields	13.2	33.5	85.9	23.4

Table 2 presents the distribution of climate-focused topics across the data sources. ‘Profitability’ and ‘Productivity’ were the most prevalent, followed by climate change adaptation and mitigation. On Twitter, CIMMYT was also extensively engaged in conversations about production processes, resource and risk management, and resistance breeding.

**Table 2:** Distribution of climate-focused topics identified in the datasets.

Climate-focused topic	Repository (%)	Scopus (%)	Twitter (%)	Overall (%)
Agroforestry	4.1	4.2	58.5	5.8
Carbon sequestration	0.4	0.8	10.4	0.9
Climate change adaptation	31.4	9.1	86.7	22.0
Climate change mitigation	31.6	9.3	87.4	22.3
Climate smart agriculture	1.8	2.2	35.6	3.0
Conservation agriculture	3.8	5.8	79.3	7.1
Crop residues	0.9	2.5	60.0	3.5
Emission reduction	0.1	0.1	15.6	0.6
Energy conservation	0.1	0.3	3.0	0.3
Mulching	3.0	2.3	59.3	4.4
Nutrient management	2.7	4.0	44.4	4.6
Nutrient use efficiency	0.4	2.5	15.6	1.9
Precision agriculture	2.0	2.3	45.9	3.5
Production	19.1	17.3	89.6	20.4
Productivity	34.2	37.3	97.8	37.7
Profitability	36.2	39.0	97.8	39.5
Resistant varieties	10.1	8.1	85.2	11.4
Resource management	16.3	17.1	85.9	18.8
Risk management	16.3	14.3	85.2	17.4
Sustainable intensification	1.9	2.1	54.8	3.6
Tolerance	1.9	6.9	50.4	5.8
Water management	11.1	15.1	75.6	15.0
Zero tillage	0.7	1.3	30.4	1.9

To unpack associations between different topics within CIMMYT’s outputs, a measure of correlation was established to identify when terms are present within the same textual object, in this case, a Repository description, a Scopus abstract or a tweet. Pearson's correlation coefficients and the asymptotic P-values were estimated on the vector with frequencies of topics for each object (descriptions, abstracts and tweets). A strong and significant correlation indicates that the terms consistently co-occur within the same body of text, whereas a negative and significant correlation indicates terms tend not to appear together. Table 3 shows the correlations between ‘Climate change’ and the remaining cross-cutting topics. The highest correlations in magnitude are seen in Repository documents, although only 5 out of 21 are significant (p < 0.10). Positive and significant correlations with ‘Climate change’ are observed for ‘Capacity development’, ‘Technology’, ‘Farming systems’, ‘Technology transfer’, and ‘Innovation’. In Scopus the magnitudes of the correlations are lower, but with more frequent significance (11 out of 21). Positive and significant correlations with ‘Climate change’ were found for ‘Capacity development’, ‘Diversification’, ‘Farming systems’, ‘Food security’, ‘Gender equity’, ‘Livelihoods’, ‘Mixed cropping’, ‘Policies’, ‘Socioeconomic development’, ‘Technology’, and ‘Yields’. Associations on Twitter are the smallest in magnitude and with only one significant: the positive association between ‘Climate change’ and ‘Enterprises’. Overall Table 3 shows that only positive associations between climate change and cross-cutting issues are significant, while negative correlations are less frequent and none are statistically significant.

**Table 3:** Correlations between ‘Climate change’ and cross-cutting topics identified in the datasets.

Cross-cutting topic	Repository	Scopus	Twitter
Capacity development	0.574***	0.034**	0.133
Diversification	0.001	0.047***	− 0.019
Enterprises	− 0.007	− 0.024	0.151*
Farming systems	0.523***	0.196***	0.062
Food security	0.002	0.059***	− 0.008
Gender equity	− 0.006	0.055***	0.013
Health	− 0.001	− 0.024	− 0.072
Innovation	0.471***	0.021	− 0.079
Livelihoods	0	0.032*	0.041
Mixed cropping	0.003	0.056***	0.007
Nematoda	− 0.004	− 0.016	− 0.018
Nitrification	0.006	0.016	− 0.017
Nutrition	0.024	− 0.003	− 0.026
Participation	0.014	0.009	− 0.045
Policies	− 0.01	0.036**	− 0.046
Services	0.021	0.017	− 0.049
Socioeconomic development	0.004	0.079***	− 0.03
Stress	0.009	0.124	− 0.006
Technology	0.563***	0.046***	0.004
Technology transfer	0.483***	0.019	− 0.09
Yields	0.009	0.124***	− 0.006

*p < 0.10, **p < 0.05, ***p < 0.01.

To understand the thematic interlinkages within the climate change-food security nexus, Table 4 shows the Pearson's correlation coefficients and the asymptotic P-values between the term ‘Climate change’ and climate-specific topics. Some variation is visible across the datasets, with Scopus showing significant correlation in most cases (17 out of 23 topics), followed by Repository items (6 out of 23), and Twitter (3 out of 23). On Scopus, among the significant associations, a higher magnitude can be found for the topics ‘Climate change adaptation’, ‘Climate change mitigation’, and ‘Risk management’. Among the significant coefficients for Repository items, a higher magnitude can be observed for ‘Productivity’, ‘Profitability’, and ‘Risk management’. On Twitter, the significant associations with climate change refer to ‘Water management’, ‘Conservation agriculture’, and ‘Resource management’. Overall, only positive correlations with climate change are significant in any dataset considered.

**Table 4:** Correlations between ‘Climate change’ and climate-focused topics identified in the datasets.

Climate-focused topic	Repository	Scopus	Twitter
Agroforestry	0.002	0.069***	− 0.041
Carbon sequestration	0.007	0.046***	0
Climate change adaptation	0.12***	0.775***	0.346
Climate change mitigation	0.118***	0.738***	0.35
Climate smart agriculture	− 0.001	0.106***	− 0.055
Conservation agriculture	0.012	0.077***	0.222**
Crop residues	− 0.007	− 0.014	− 0.042
Emission reduction	− 0.005	0.071***	− 0.025
Energy conservation	0.002	0.014	0
Mulching	− 0.018	− 0.019	− 0.04
Nutrient management	− 0.004	0.127***	− 0.003
Nutrient use efficiency	− 0.001	0.015	− 0.01
Precision agriculture	0	0.053***	− 0.074
Production	0.543	0.088***	− 0.072
Productivity	0.789***	0.07***	− 0.054
Profitability	0.768***	0.051***	− 0.04
Resistant varieties	− 0.021	0.011	0.063
Resource management	0.498***	0.173***	0.16*
Risk management	0.556***	0.263***	− 0.063
Sustainable intensification	0.012	0.03*	− 0.043
Tolerance	0.006	0.06***	− 0.042
Water management	0.017	0.149***	0.241**
Zero tillage	− 0.002	− 0.015	0.036

*p < 0.10, **p < 0.05, ***p < 0.01.

### Hyperlink analysis: mapping the dissemination of climate research outputs

**Figure 3:**
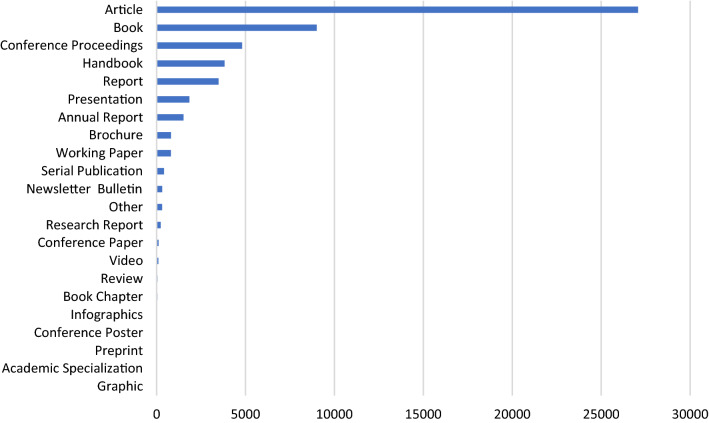
Frequency of URLS by publication type.

Of the 2,463 climate-related research outputs collected in CIMMYT’s Repository, our algorithm generated non-ambiguous results for 2,263 items, for a total of 55,151 web pages pointing to them. More than 10 thousand unique domains and over 150 countries were identified. Figure 3 breaks down the frequency of URLs by type of publication, as identified by Repository metadata. It shows that scientific research such as articles and books were the most frequently disseminated outputs, followed by outreach materials such as handbooks, reports and presentations.

**Figure 4:**
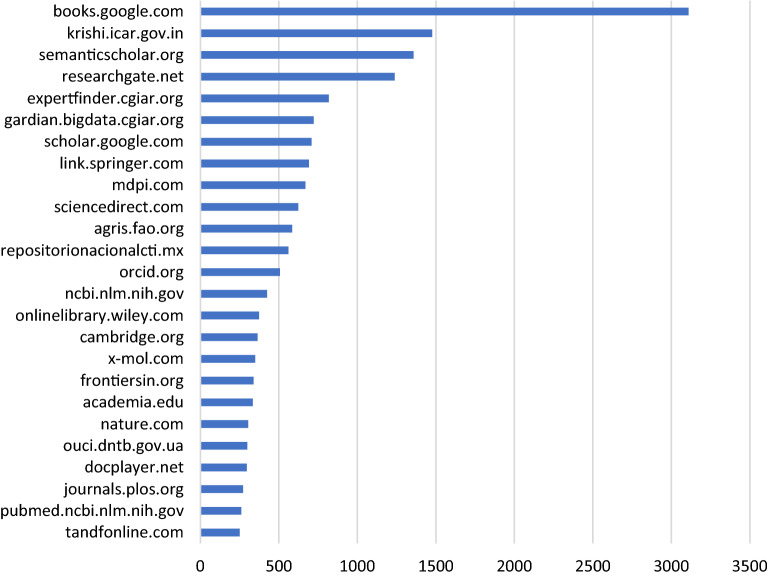
Frequency of top 25 domain names distributing CIMMYT’s climate-related outputs (CIMMYT website and repository excluded).

Figure 4 shows the 25 most frequent domain names detected in the analysis. Books stored in Google Books contain more than 3,000 references to CIMMYT’s climate-related outputs, and most frequent domains pertain to academic publishers, reflecting both the scientific activity of CIMMYT as well as citations in external papers. However, government partners such as the Agricultural Knowledge Resources and Information System Hub for Innovations (KRISHI) of the Indian Council of Agricultural Research (ICAR), and international organizations like the Food and Agriculture Organization (FAO) are also featured among key multipliers of CIMMYT’s research.

**Figure 5:**
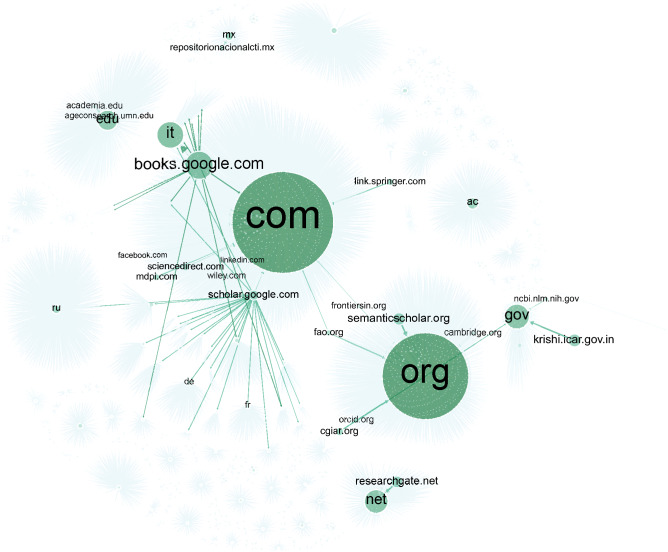
Network visualization of Top-Level Domain distribution.

Figure 5 presents a network visualization of the top-level domains (TLD) extracted from the URLS and their respective top domains. TLDs are split into two types: generic TLDs that do not correspond to a particular country or region, such as .com and .org.; and country-code TLDs that represent a geographical location, such as .uk (United Kingdom) or .mx (Mexico). The TLD distribution approximates the geographic distribution and the types of entities picking up and disseminating the organization’s outputs^41^. It shows that CIMMYT reaches a variety of institutions, from commercial publishers and private companies to civil society organizations, universities, and governments in several countries.

The network shows that most sites sharing CIMMYT’s knowledge products are “.com”, a TLD generally used by commercial institutions, followed by “.org”, often used by non-profit organizations. Three other TLDs well represented in the graph are “.gov” (used by government bodies), “.edu” (reserved for educational institutions) and “.net” (generally used by online service providers). The most frequent domains with “.com” TLD indicate that, despite a prevalence of commercial publishers, CIMMYT content is also disseminated across social networking platforms such as LinkedIn, and Facebook. We detected CIMMYT’s presence in 13,630 URLS from 2,543 unique “.com” domains. A similar analysis of “.org” domains detected a total of 12,467 URLS from 1,334 domains. Here we see again many academic repositories, but also partners and funders such as the CGIAR and FAO.

**Figure 6:**
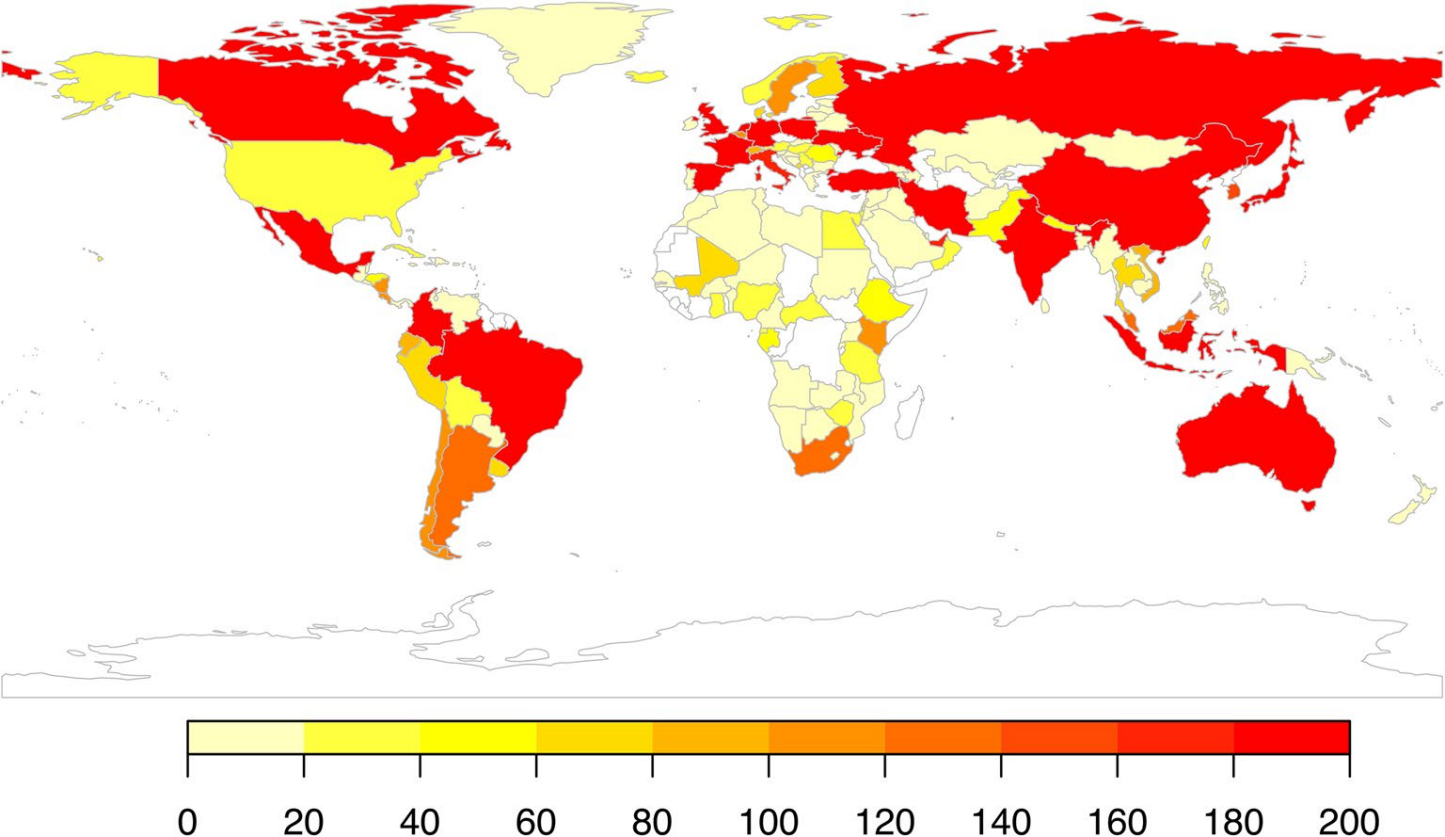
Geographical distribution based on country-code TLD. Countries colored in red contain TLDs with more than 180 URLs; orange shades are between 60 and 180; yellow are between 1 and 60. This map was created in R, version 4.1.2 (2021–11-01), link: https://cran.r-project.org/.

Figure 6 presents the geographical distribution of the country-code TLDs. The countries colored in red contain TLDs with more than 180 URLs; the orange shades are between 60 and 180; and yellow are between 1 and 60. A limitation must be noted in this particular metric: in general U.S.-based institutions do not use their country specific TLD (.us), normally opting for generalist TLDs, which has resulted in an under-representation of U.S.-based domains. Nevertheless, and taking that into account, it is possible to see that CIMMYT’s climate change related outputs have been distributed to more than 150 countries across both the Global North and South. Domains in North America, Australia, and Europe have frequently disseminated CIMMYT research, but also in South and Southeast Asia, and Latin America.

### Network analysis: locating CIMMYT within climate dialogue exchanges

**Figure 7:**
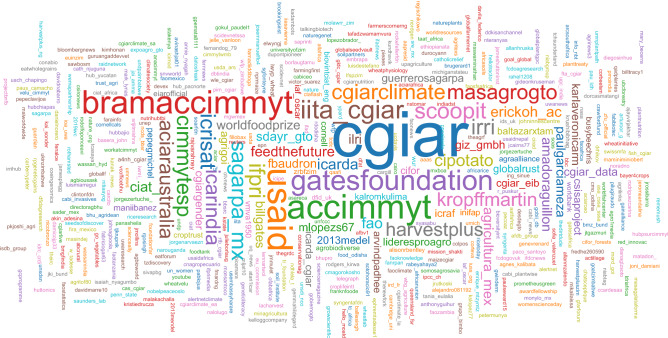
Word cloud of account mentions identified in tweets containing either @CIMMYT or #CIMMYT.

One of the central features of Twitter is the possibility of interaction among users through the @mention, when an account is tagged and notified, giving it the ability to engage in direct dialogue. Mentions are part of Twitter’s discursive affordances and serve mainly to acknowledge, draw attention or engage in a public conversation with a particular user^42^. Figure 7 shows a word cloud for the accounts mentioned in the Twitter corpus (CIMMYT’s main English account excluded), in which the main account of the CGIAR consortium (@cgiar)—CIMMYT’s umbrella organization—is the most prevalent, followed by American bilateral development agency USAID (@usaid), CIMMYT’s Spanish account (@accimmyt), the institution’s Director General a.i (@bramaccimmyt), and the philanthropic Bill & Melinda Gates Foundation (@gatesfoundation).

**Figure 8:**
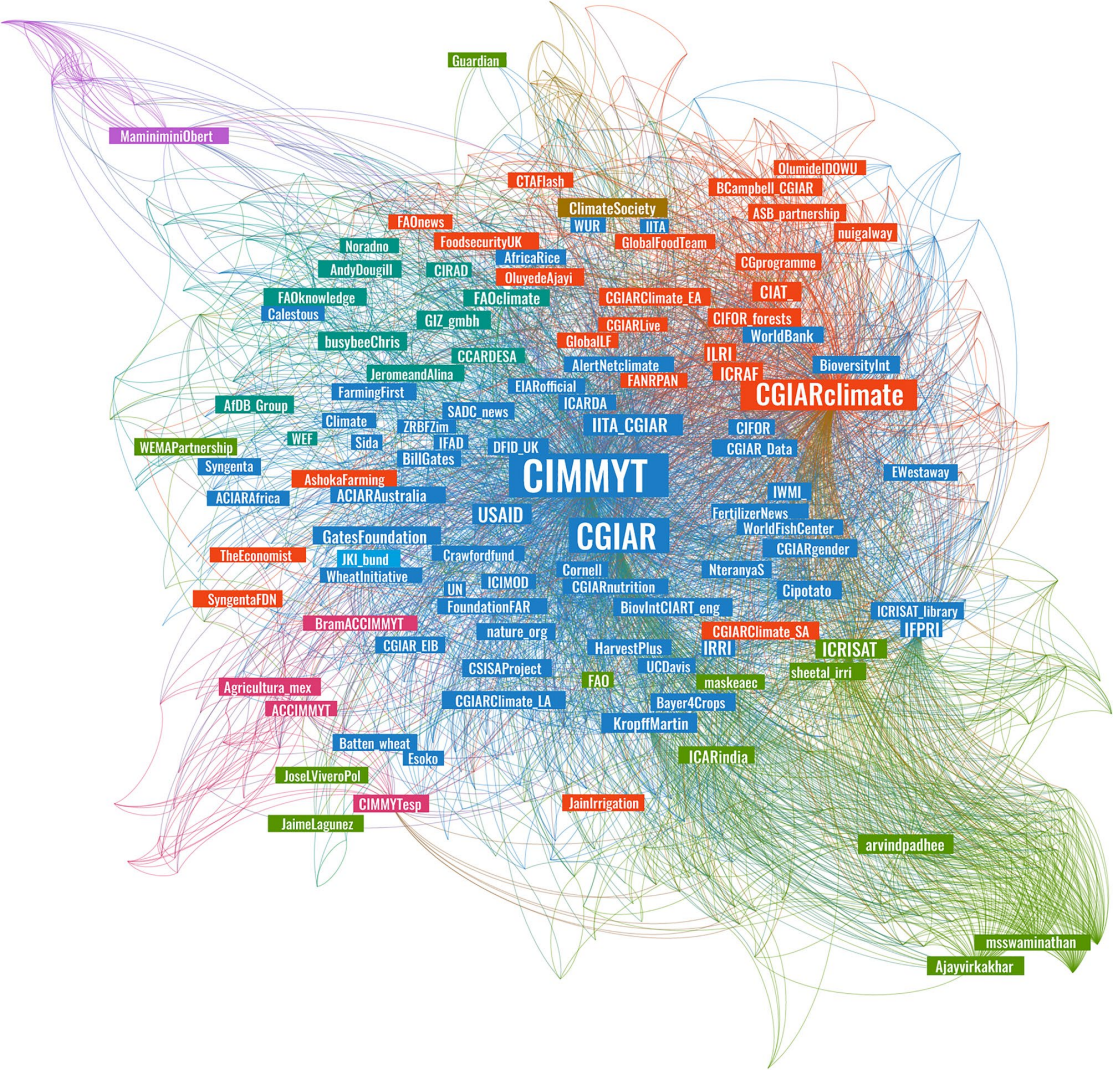
Twitter mentions network (Parameters: force-directed graph, with node size partitioned as Weighed in-Degree, colored by modularity class. Nodes restricted to those mentioned at least five times. Nodes = 704 accounts, edges = 4,755 connections).

Focusing on the subset of tweets about climate change, we assessed the dynamics between tweets by CIMMYT and tweets that mentioned the organization either through @CIMMYT or #CIMMYT. The mentions network comprises directed links that indicate one user has referred to another user in a tweet^36^. The complete network of mentions derived from this subset contained more than 2,700 unique users, connected almost 8,000 times. To uncover key actors within this broad network, the analysis considered only accounts that were mentioned at least five times (i.e., in-degree distribution). This criterion reduced the number of nodes to 704, with 4,755 connections between them, making the network denser, with nodes connected on average to 6.8 other nodes. The resulting visualization is presented in Fig. 8.

The force-directed spatialization provides a visual interpretation of the dynamics between actors in the network by drawing linked nodes closer together while pushing apart unrelated nodes. As nodes as positioned according to their connectivity, this enables identifying central and peripheral nodes according to their location, as well as nodes with frequent interlinkages. The sizes of the labels have been set according to the node’s in-degree centrality, which measures the frequency with which accounts have been mentioned in this dataset. The largest and most central nodes belong to CIMMYT’s main account (@CIMMYT) followed by the CGIAR Research Program on Climate Change Agriculture and Food Security (CCAFS) (@CGIARclimate), under which much of the agricultural adaptation activity of CGIAR centers was implemented.

The modularity algorithm identified five major communities, with nodes colored accordingly. The Louvain method^39^ determines the level of homogeneity within a network by identifying communities comprising groups of nodes that interact more frequently with each other than they do with others. In this dataset, each community represents groups of Twitter profiles that more frequently appear within the same tweets, indicating the dynamics of interactions in the network. A modularity coefficient of 0.137 (on a scale from − 1 to + 1) suggests a heterogeneous network in which communities show some level of interconnectivity, but where nodes mostly interact beyond their clusters and with other nodes in the network.

The largest group is in blue, representing 42% of the network and featuring CIMMYT as the key actor. Within this cluster and close to CIMMYT we also find funding organizations such as USAID, the UK’s former bilateral development agency, the Department for International Development (@DFID_UK), the UN’s International Fund for Agricultural Development (@IFAD) and the Gates Foundation, indicating frequent interactions between these accounts.

**Figure 9:**
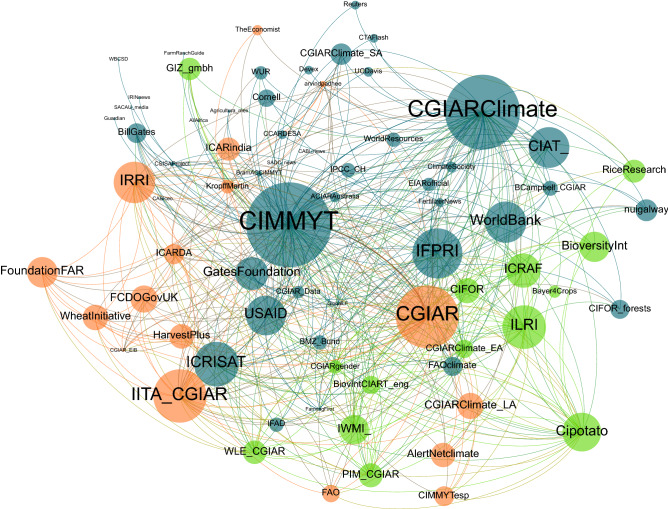
Top 75 influential nodes in Twitter mentions network (Parameters: force-directed graph, with node size partitioned as Eigenvector Centrality, colored by modularity class. Nodes = 75 accounts, edges = 518 connections).

Figure 9 shows the 75 most mentioned accounts sized according to their Eigenvector centrality, a metric that establishes the most influential nodes in the network. Eigenvector centrality is a common metric for social media analysis that measures the influence of actors within a network by considering not only how many connections a node has, but also the centrality of the nodes that it is connected to^43^. The importance of a node is measured by how much it is connected to other important nodes in the network, and typically on social media, actors with high eigenvector centrality are important centers of attention. The most influential nodes identified by this metric are CIMMYT (@CIMMYT) and CCAFS (@CGIARclimate). The networks display a visual representation of the dynamics of information exchanges related to climate change in which CIMMYT participates in, where it is well positioned to connect actors and influence information flows.

**Figure 10:**
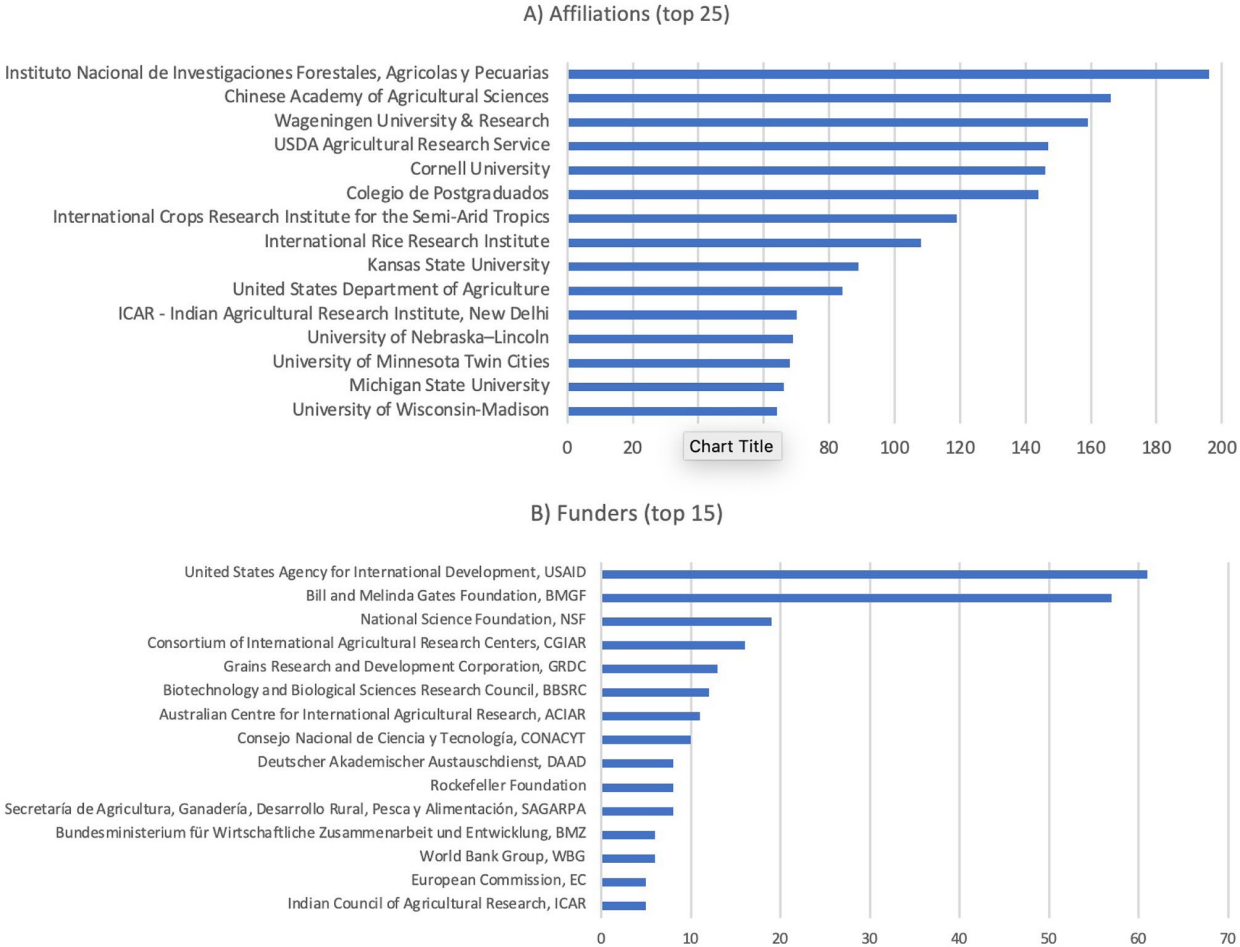
Most frequent institutional affiliations (**A**) and funders (**B**) identified in CIMMYT-affiliated climate-focused publications indexed by Scopus.

The Scopus dataset contained author affiliation metadata that can be assessed for networks of scientific collaboration. This analysis was performed on 1,289 publications classified under the climate-focused topics that identified more than 500 unique institutions. Figure 10 presents most frequent institutional collaborations and funders of CIMMYT’s climate research.

**Figure 11:**
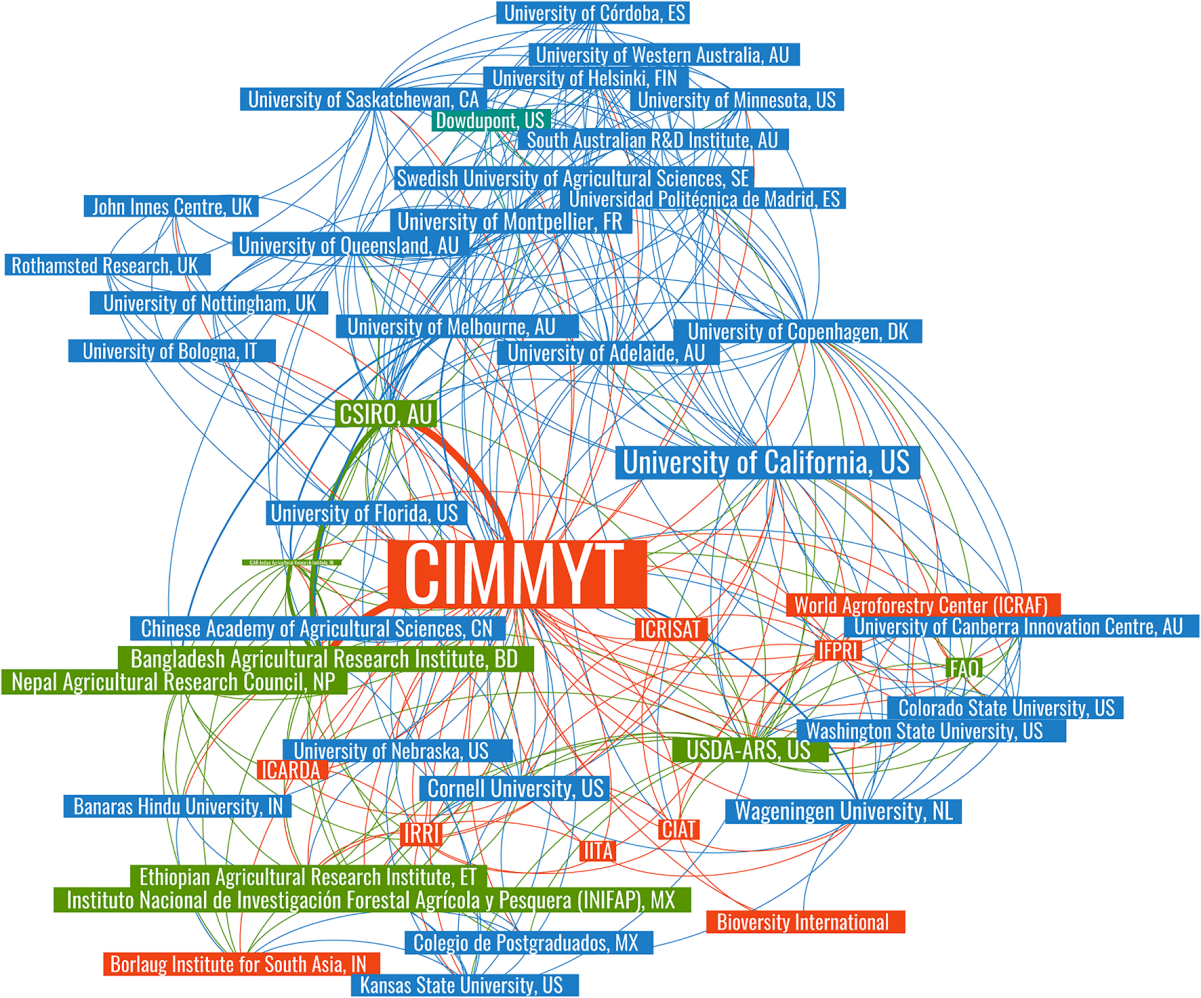
Network of scientific cooperation (Parameters: force-directed graph, with node size partitioned by total link strength. Nodes restricted to those connected at least 50 times. Nodes = 66 institutions, edges = 425 connections).

As institutional affiliations are not standardized in the Scopus index, the dataset was manually checked and cleaned for duplicates—different departments or regional offices were merged into their top-level institution. Figure 11 presents a network visualization for the top collaborations between CIMMYT and research organizations, identified from the institutional affiliations of authors in Scopus-indexed publications. Node colors are coded by organization type: Universities (in blue, representing 58% of the network), CGIAR Research Centers (in red, comprising 20% of the network), and public research institutions (in green, representing 20%). There is also a private sector partner (DowDuPont) identified in teal blue. The most frequent scientific collaborations occurred with the University of California, the Commonwealth Scientific and Industrial Research Organization (CSIRO), the Indian Council of Agricultural Research (ICAR), the U.S. Department of Agriculture’s Agricultural Research Service (USDA-ARS) and the International Rice Research Institute (IRRI).

## Discussion

Using CIMMYT’s food production-climate change nexus activities as an example, this study proposes a novel framework to explore the production and diffusion of scientific research outputs through comprehensive analyses of online sources that repurpose digital objects and their technical and discursive affordances to assess issue formation and dynamics. It contributes to the evolution of bibliometric analysis, which has been expanding beyond measuring the impact of academic outputs to include systematic, machine-driven thematic reviews^14,44,45^, and aims to fill a gap in research intended to assess scientific reach beyond networks of researchers and research institutions^46^.

The text mining analyses indicate converging and diverging trends across the data sources. The geographical focus of CIMMYT’s climate research has centered on three main countries: Mexico, India, and Ethiopia. ‘Farming systems’ and ‘Food security’ were the most frequent cross-cutting topics, though ‘Innovation’, ‘Technology transfer’, ‘Yields’ and ‘Stress’ were also common themes. ‘Profitability’, ‘Productivity’, ‘Climate change adaptation’ and ‘Climate change mitigation’ were the most frequent climate-focused topics, though on Scopus, greater attention was paid to ‘Production’, ‘Resource management’ and ‘Water management’. While institutional conversations involving CIMMYT on social media have addressed the effects of climate change on the livelihoods of rural people, this connection was not reflected in the institution’s scientific and non-scientific knowledge products, which focused on research around the development of agricultural technologies and practices to support food production, cope with climate impacts, and mitigate climate forcing.

Variability among the correlations between climate change and cross-cutting topics was also observed. While ‘Technology Transfer’ and ‘Innovation’ were frequently associated to climate change in Repository items, indicating CIMMYT’s strategic interest in proposing solutions to climate-sensitive agriculture, this was not reflected in neither Scopus nor Twitter. In CIMMYT’s social media conversations, associations between climate change and climate-focused topics showed a stronger emphasis on water management and conservation agriculture; in Repository materials, climate change appears more alongside productivity and profitability, and in indexed scientific papers, mitigation and adaptation present the strongest associations.

The hyperlink analysis shows that the uptake of climate science generated through CIMMYT’s research has been strongest on academic and research platforms, but is also evident on social media, government and international organization websites from both the Global North and South. Scientific articles and books are not only the most produced research outputs, but are also the most disseminated, despite the existence of many other non-scientific products. This presents a potential for CIMMYT to work across audiences by creating opportunities for exchanges beyond the scientific community and achieving a bigger impact at the policy level.

As a research technique, finding hyperlinks through the entire web is very ambitious; dedicated projects exist with the goal of building updated and comprehensive hyperlink graphs, such as the Web Data Commons project^47^. Coverage of the web remains a major challenge, as the most complete collection of web pages is provided by private entities such as Google, Yahoo, or Microsoft, which monetize their services. While public web archives exist—e.g. WayBack Machine^48^ or Common Crawl^49^—they might miss a good number of web pages, resulting in limited outcomes^50^. Building a dedicated search engine could solve the coverage issue but would require big data infrastructures that go beyond ordinary technology. Web scraping the major search engines was the viable alternative to balance between coverage and resource limitations, with further studies required to refine the data extraction algorithm.

Social network analysis has been employed extensively for understanding a network's structure, operation, and dynamics^51,52^. Issue networks are characterized as social structures that arise around specific issues or policy concerns and consist of a configuration of social ties among interdependent players^53^. Studies focused on social media network formations recognize platforms like Twitter as important spaces for information exchange, debate, and opinion formation on a range of issues, with the network structures formed through online debate affecting how attitudes evolve over time^36^. CIMMYT’s place in the network of climate-related conversations where it is mentioned shows that the organization actively interacts with diverse actors from scientific, development and public policy communities, bringing forward climate-sensitive agriculture to the public debate. A limitation to note is the dataset’s bias towards the institution, as the tweet collection was based on CIMMYT mentions. Further research is recommended to collect and analyze issue-based social media data to assess CIMMYT’s influence in a broader context of climate discussions.

The network of scientific cooperation identified through Scopus data also supports CIMMYT’s pursuit of co-production and inter-institution relationship building, as the organization has collaborated with hundreds of academic and research institutions to enhance climate science.

## Conclusion

Our results complement a growing body of research that shows the potential of social network platforms, search engines and other web-based sources in identifying issue networks and measuring public awareness. The web analytics framework proposed in this paper could be a useful approach for research for development organizations to assess the extent of their knowledge production, dissemination, and influence from an integrated perspective that maps both the scientific landscape and public engagement.

Results show that CIMMYT has consistently increased its focus on climate-sensitive research, and that it effectively engages in co-creation, exchanges, and diffusion of climate science at global and local levels. Historical textual data shows a significant shift towards a research agenda focused on sustainability and climate responsive practices. Hyperlink data reveals the amplification of CIMMYT’s research outputs across thousands of websites and platforms worldwide. Networks of social media interactions and scientific collaborations indicate the institution’s extensive efforts to engage with and beyond the scientific community to contribute to the climate debate.

The multimodal analysis also shows discrepancies between institutional research objectives (represented by Repository and Scopus data) and actual influence (represented by the hyperlink analysis and Twitter conversations), particularly with regards to establishing a prominent role in the science-policy interface. Our findings can inform strategic planning and decision-making to address this diversion, potentially through the enhancement of targeted advocacy that maps policy actors, proposes specific outreach, and adapts scientific outputs into more accessible products.

## Supplementary Information

Supplementary Information.

## Data Availability

The datasets generated and/or analyzed during the current study are available upon request to corresponding authors.
