# Supplementary material for: Digital artifacts reveal development and diffusion of climate research
Source: Sci Rep. 2022 Aug 19;12:14146. doi: 10.1038/s41598-022-17717-8 (PMC9391477; doi:10.1038/s41598-022-17717-8)
Supplement: Supplementary file 1 — Supplementary Information. [file 41598_2022_17717_MOESM1_ESM.pdf]

Supplementary Information

Digital artifacts reveal development and diffusion of climate research

Bia Carneiro

Giuliano Resce

Tek Bahadur Sapkota

Contact information:

Bia Carneiro: biacarneiro@ces.uc.pt

Tek B. Sapkota: T.Sapkota@cgiar.org

This PDF includes:

Supplementary Information A – Descriptives from CIMMYT Repository

Supplementary Information B – Country detection by source

Supplementary Information C – Time trends for cross-cutting topics by source

Supplementary Information D – Time trends for climate-focused topics by source

Supplementary Information A – Descriptives from CIMMYT Repository

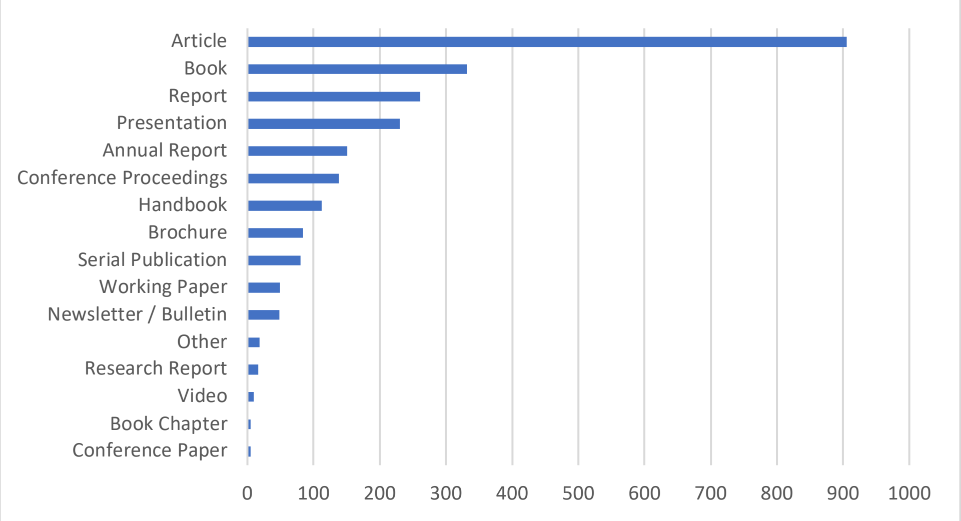

Figure A1. Repository publication types identified in climate-related knowledge products available in the CIMMYT Repository.

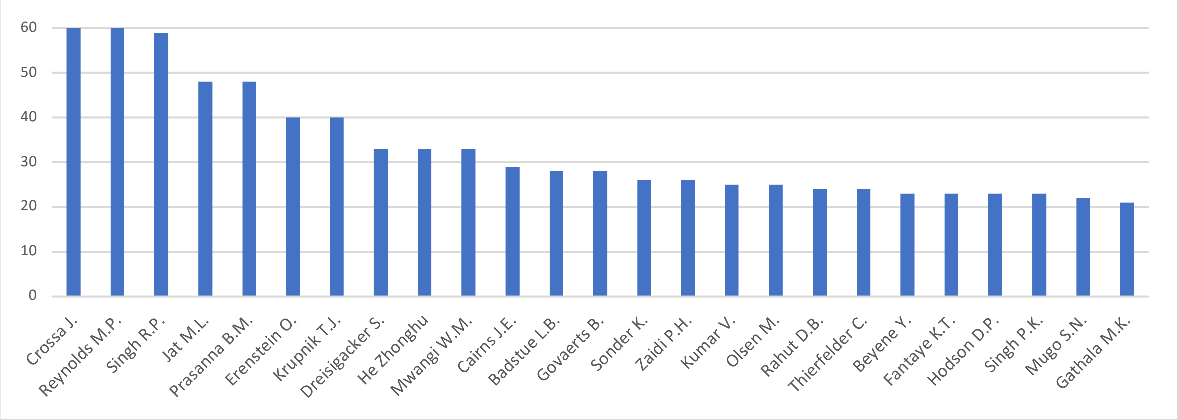

Figure A2. Top 25 authors identified in climate-related knowledge products available in the CIMMYT Repository.

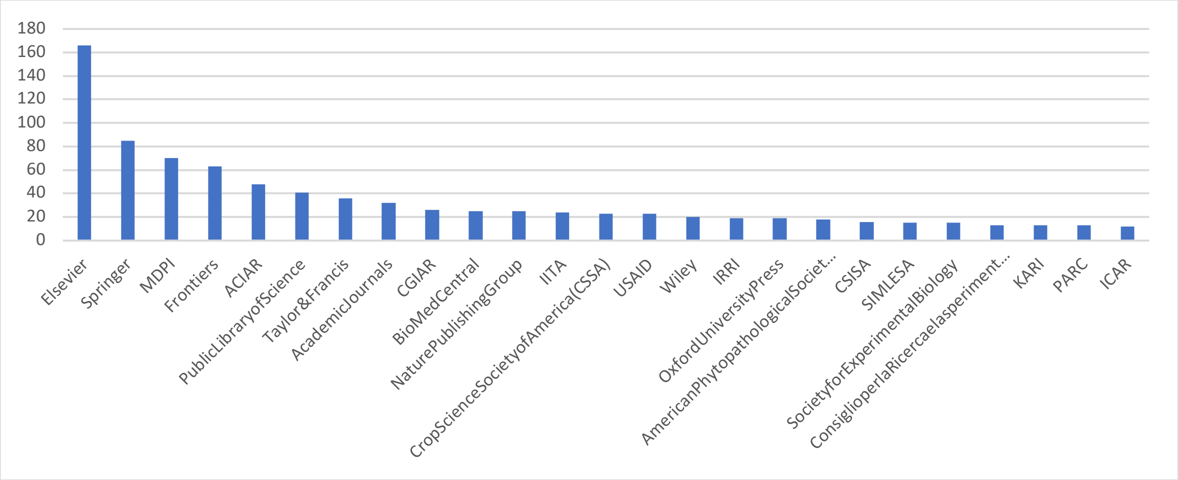

Figure A3. Top 25 publishers identified in climate-related knowledge products available in the CIMMYT Repository.

Supplementary Information B – Country detection by source

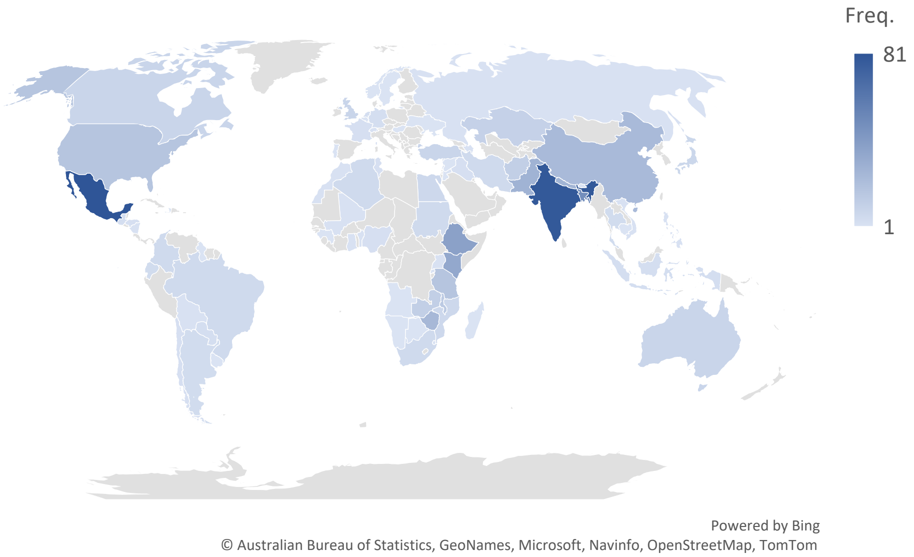

Figure B1. Frequency of countries identified in climate-related knowledge products available in the CIMMYT Repository. Map generated on Microsoft Excel for Mac, Version 16.64, used with permission from Microsoft.

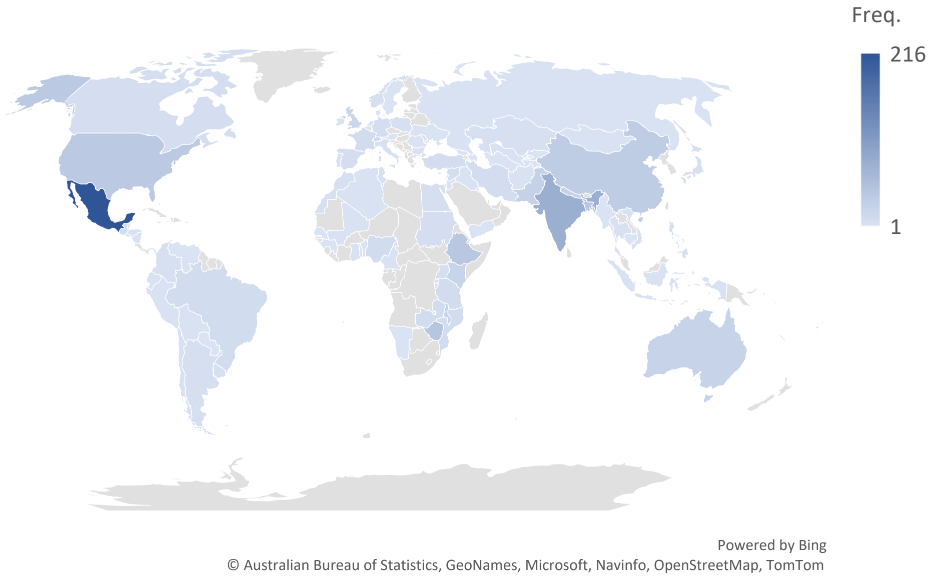

Figure B2. Frequency of countries identified in CIMMYT's climate-related publications indexed in Scopus. Map generated on Microsoft Excel for Mac, Version 16.64, used with permission from Microsoft.

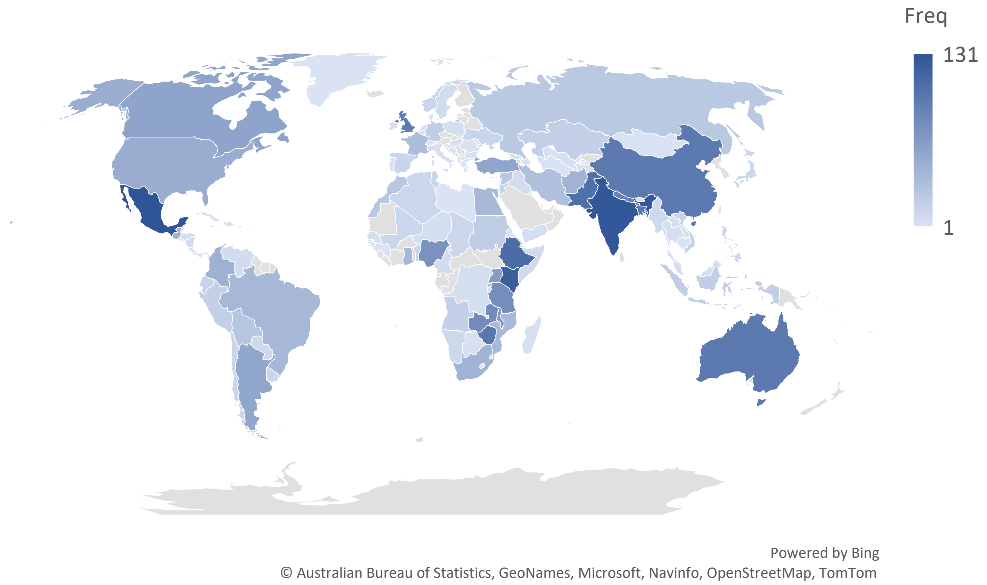

Figure B3. Frequency of countries identified Twitter, for Tweets containing either @CIMMYT or #CIMMYT. Map generated on Microsoft Excel for Mac, Version 16.64, used with permission from Microsoft.

Supplementary Information C – Time trends for cross-cutting topics by source

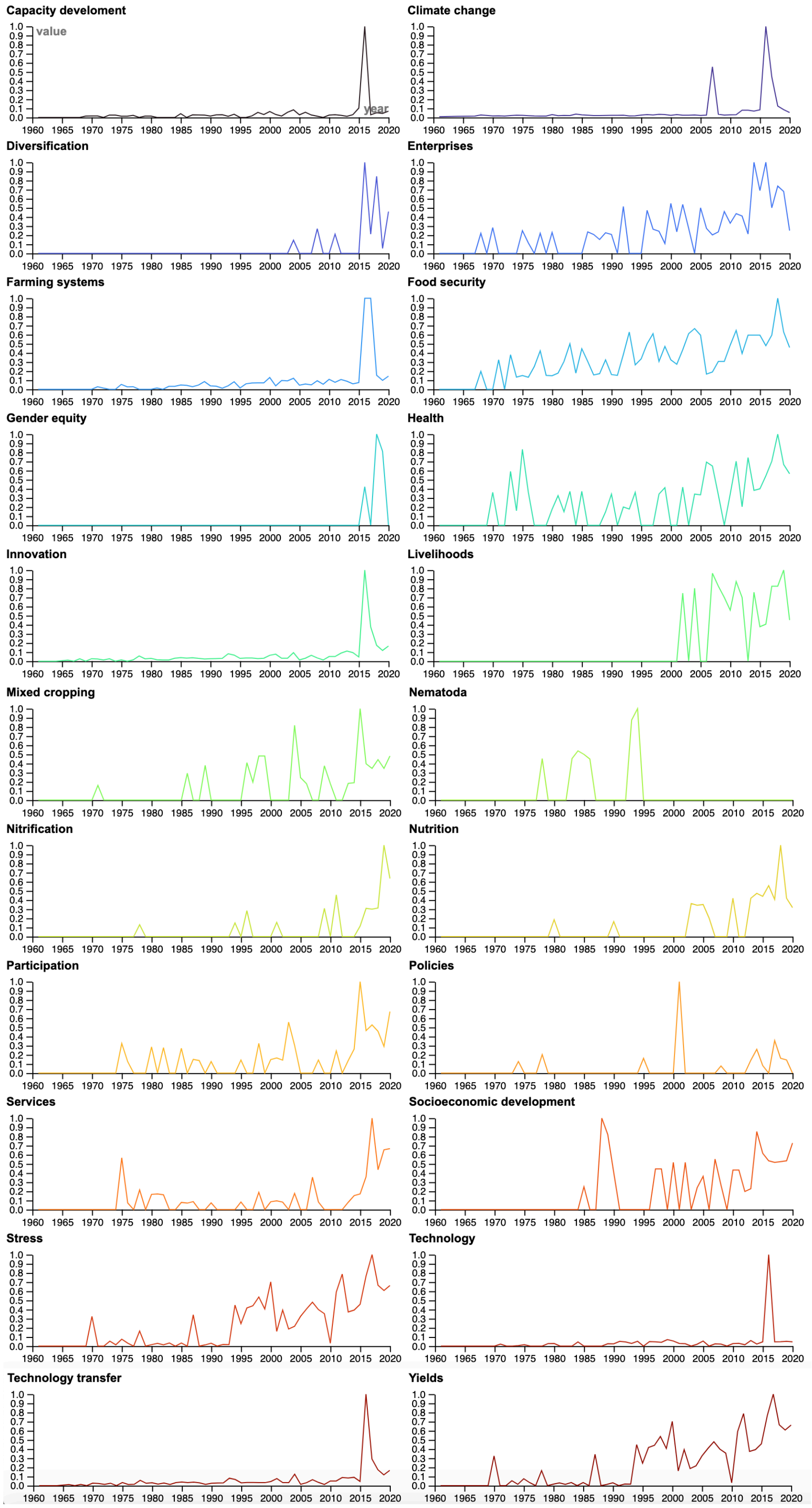

Figure C1. Timelines for the prevalence of cross-cutting topics identified in climate-related knowledge products available in the CIMMYT Repository (topics normalized on scale from 0.0-1.0).

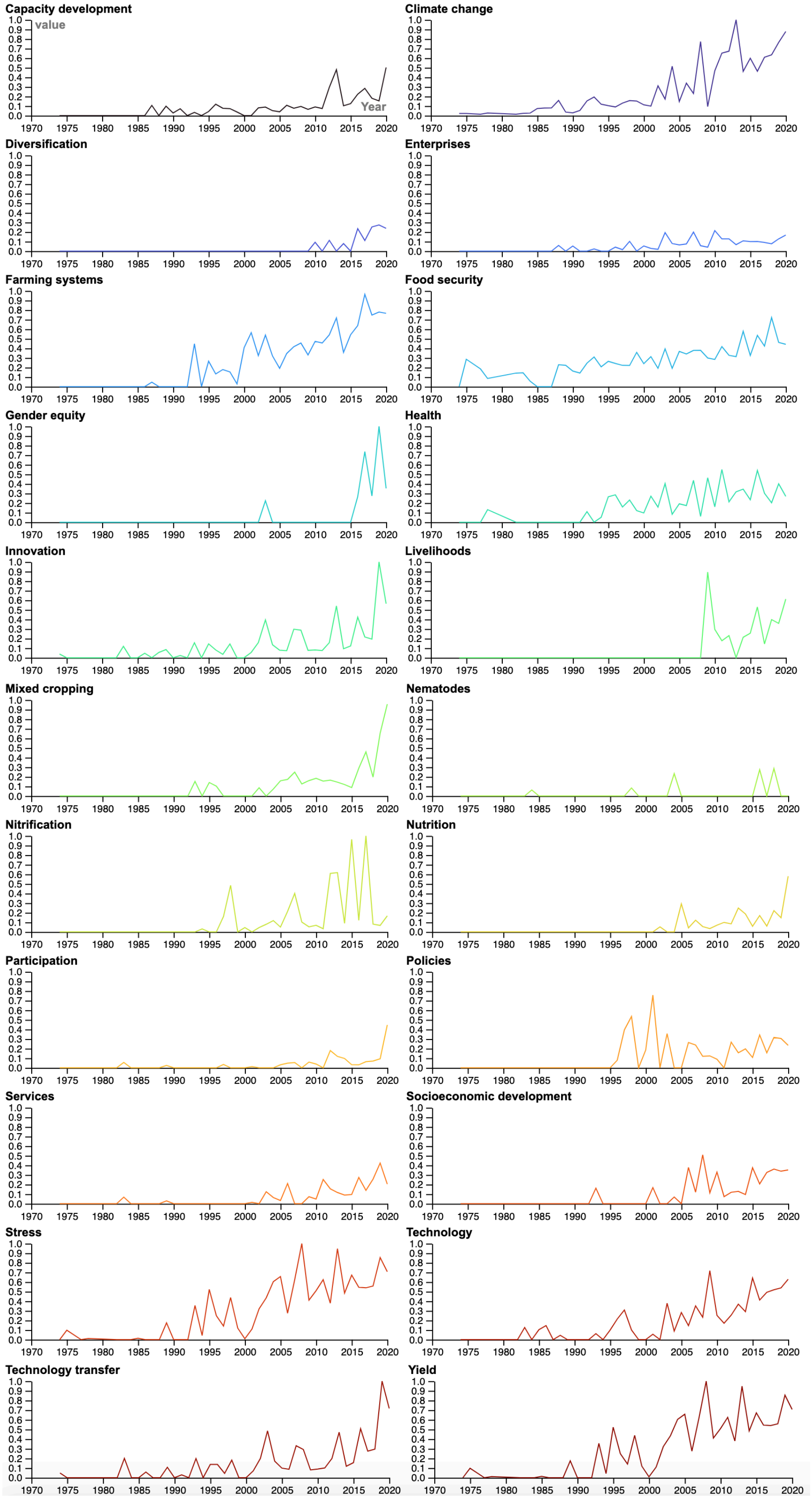

Figure C2. Timeline for the prevalence of cross-cutting topics identified in CIMMYT-affiliated publications indexed by Scopus (topics normalized on scale from 0.0-1.0).

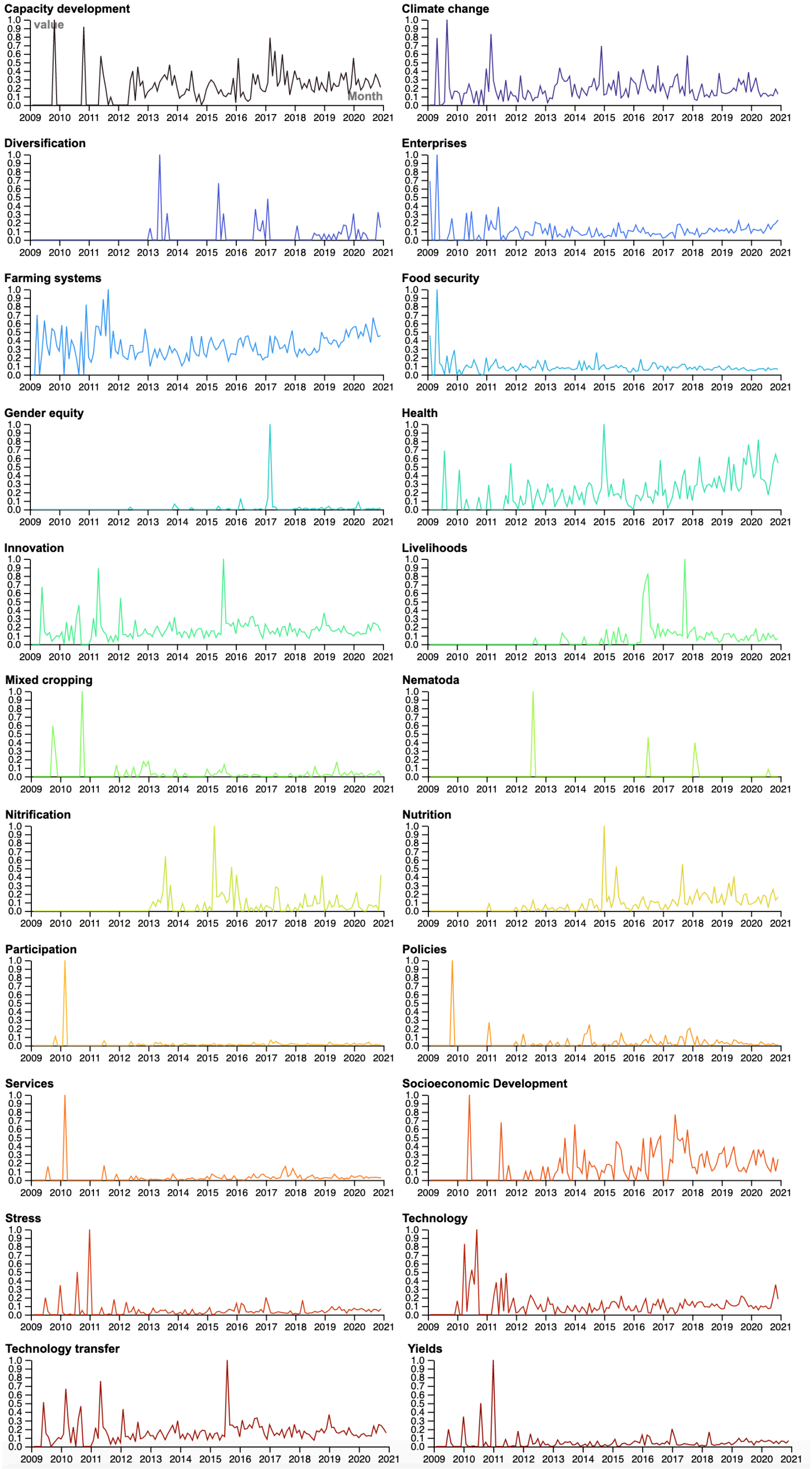

Figure C3. Timeline for the prevalence of cross-cutting topics identified on Twitter, for Tweets containing either @CIMMYT or #CIMMYT (topics normalized on scale from 0.0-1.0).

Supplementary Information D – Time trends for climate-focused topics by source

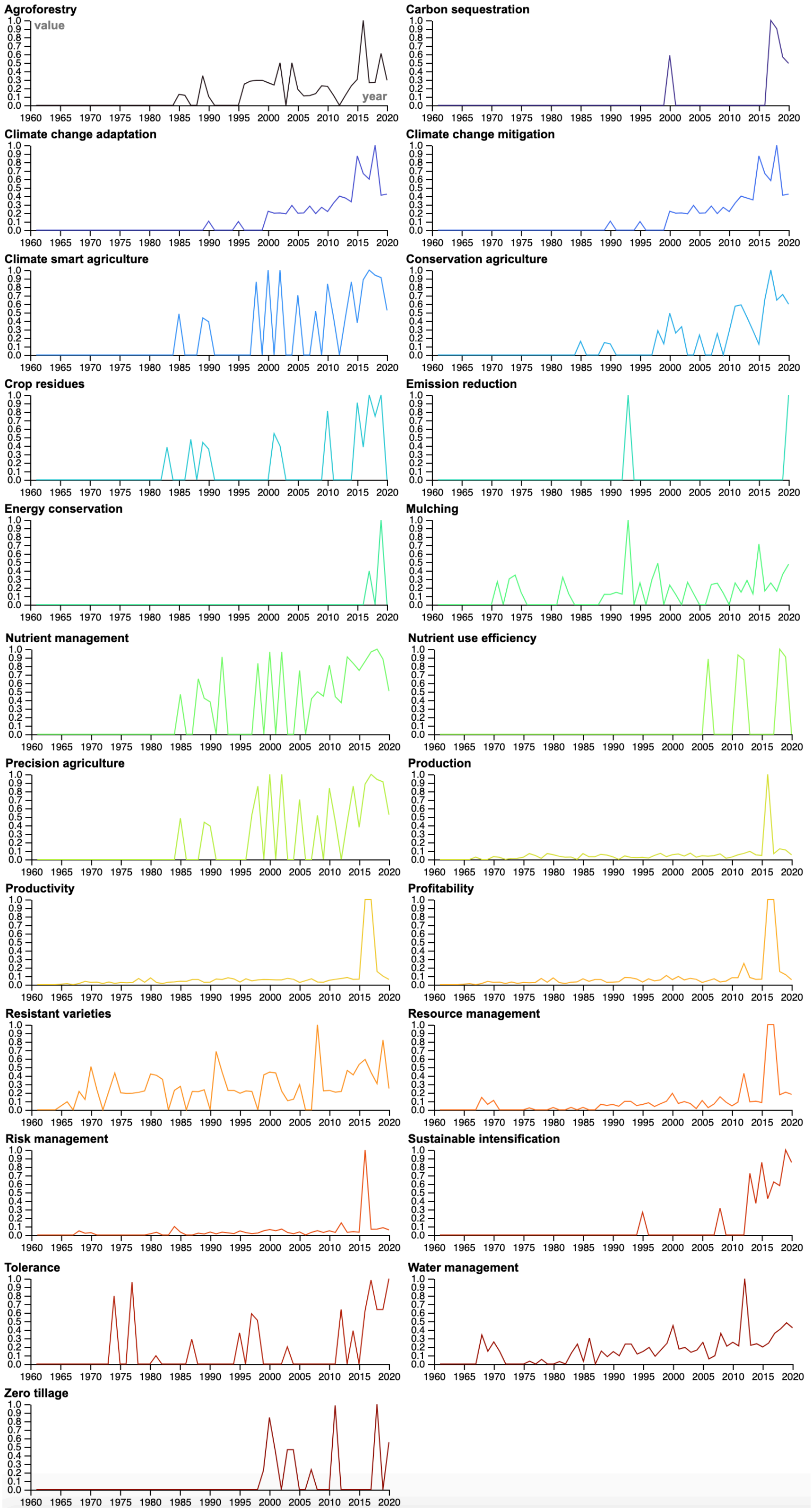

Figure D1. Timeline for the prevalence of climate topics identified in climate-related knowledge products available in the CIMMYT Repository (topics normalized on scale from 0.0-1.0).

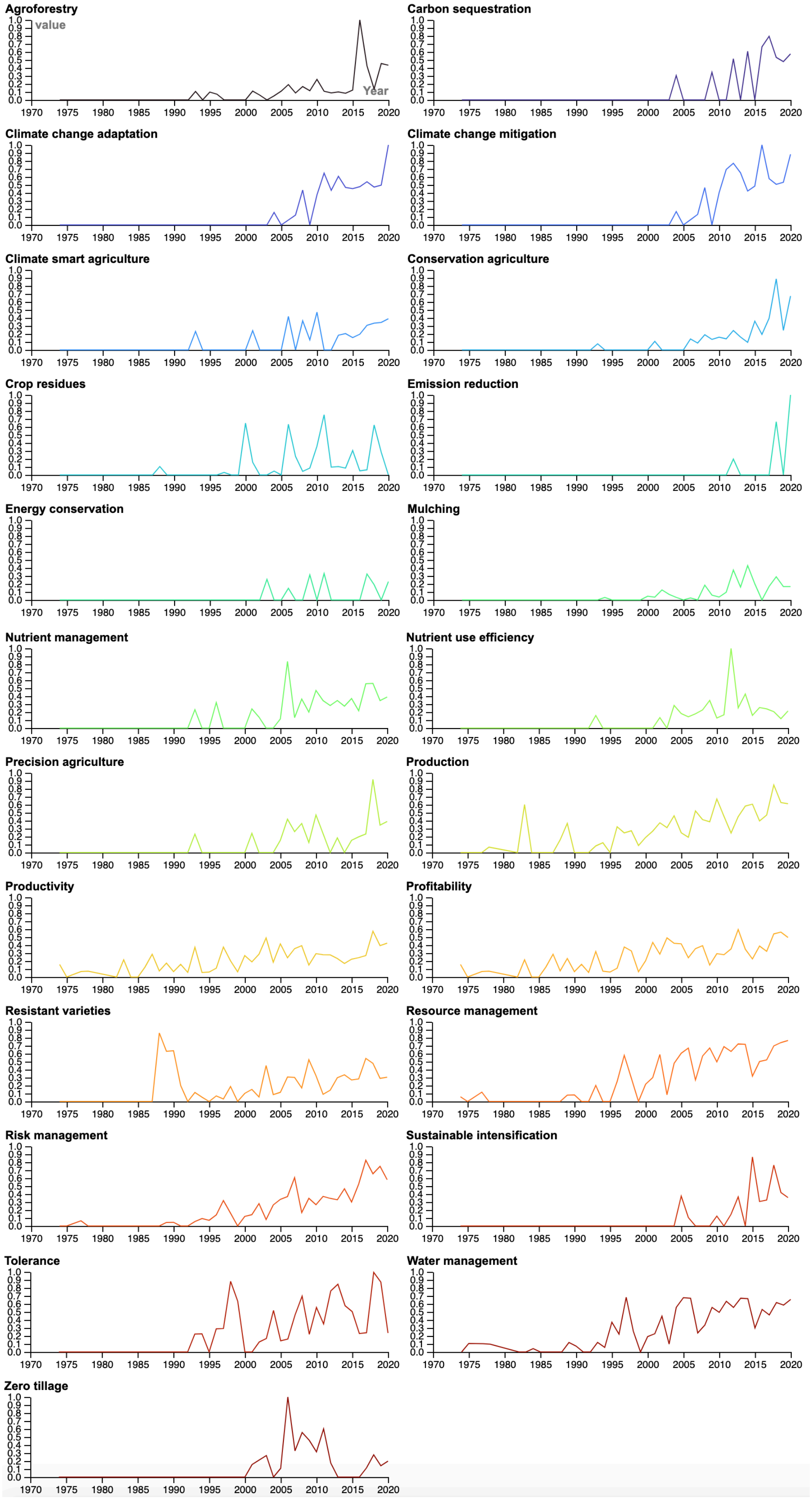

Figure D2. Timeline for the prevalence of climate topics identified in CIMMYT-affiliated publications indexed by Scopus (topics normalized on scale from 0.0-1.0).

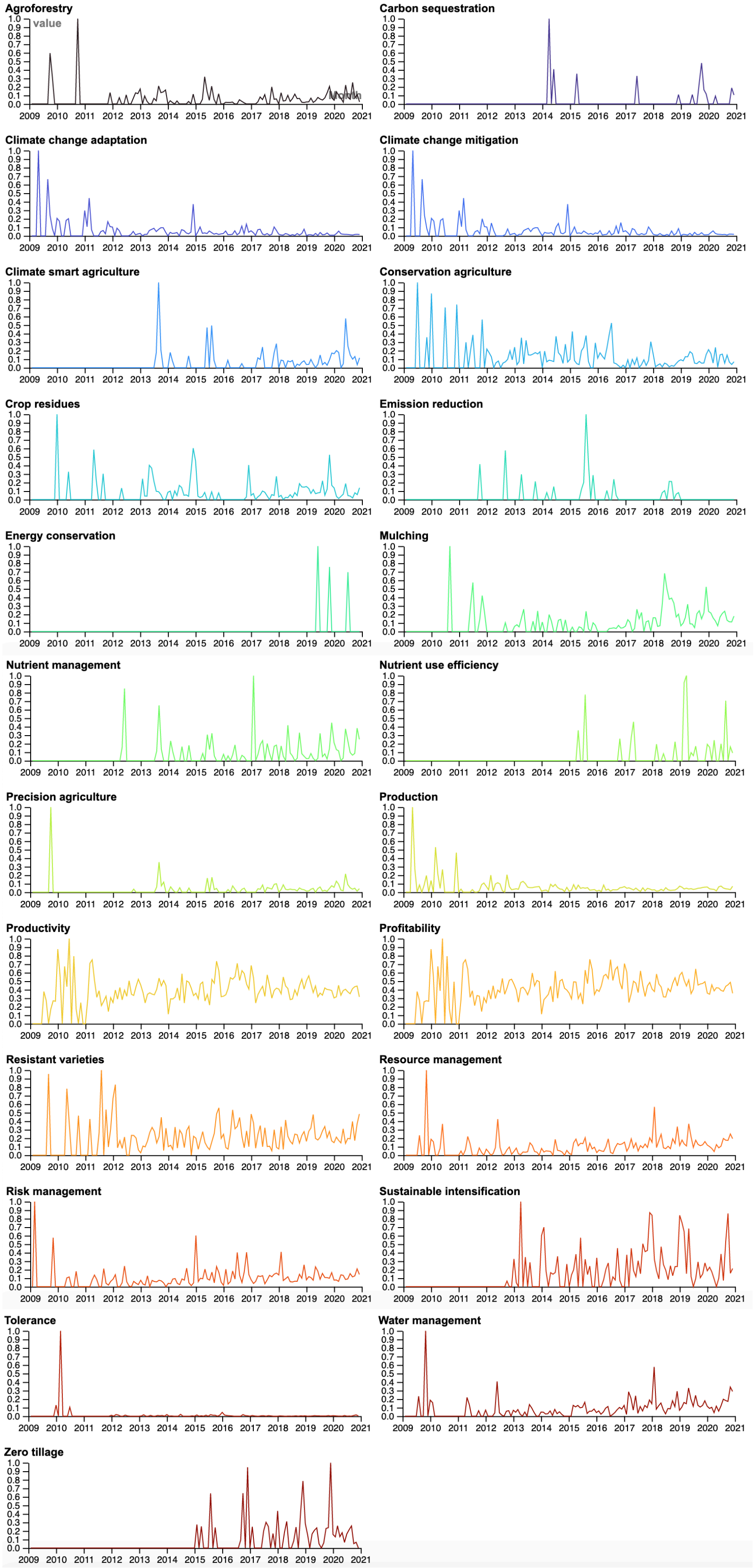

Figure D3. Timeline for the prevalence of climate topics identified on Twitter, for Tweets containing either @CIMMYT or #CIMMYT (topics normalized on scale from 0.0-1.0).
